# The Role of Fat Mass and Obesity-Associated (FTO) Gene in Non-Small Cell Lung Cancer Tumorigenicity and EGFR Tyrosine Kinase Inhibitor Resistance

**DOI:** 10.3390/biomedicines13071653

**Published:** 2025-07-07

**Authors:** Aayush Rastogi, Rong Qiu, Rachel Campoli, Usama Altayeh, Sarai Arriaga, Muhammad J. Khan, Subaranjana Saravanaguru Vasanthi, Robert Hillwig, Neelu Puri

**Affiliations:** 1 Department of Biomedical Sciences, University of Illinois College of Medicine at Rockford, Rockford, IL 61107, USA; arasto5@uic.edu (A.R.); rqiu5@uic.edu (R.Q.); rcamp29@uic.edu (R.C.); ualta@uic.edu (U.A.); sarria9@uic.edu (S.A.); mkhan@mcw.edu (M.J.K.); ssara24@uic.edu (S.S.V.); 2 Department of Health Sciences Education, University of Illinois College of Medicine at Rockford, Rockford, IL 61107, USA; rhillwig@uic.edu

**Keywords:** fat mass and obesity-associated gene (FTO), EGFR TKI resistance, non-small cell lung cancer (NSCLC), N6-Methyladenosine (m6A), late stage, smokers

## Abstract

**Background/Objectives**: The fat mass and obesity-associated (FTO) protein demethylates nuclear N6-Methyladenosine (m6A) on mRNA, facilitates tumor growth, and contributes to therapeutic resistance in multiple cancer types. Recent evidence demonstrates several roles of FTO in tumorigenesis. In this study, we seek to explore the role of FTO in non-small cell lung cancer (NSCLC) tumorigenicity and its relationship with epidermal growth factor receptor (EGFR) tyrosine kinase resistance. **Methods**: We performed qPCR, immunoblotting, viability assays, migration assays, and ATP assays to investigate the functions of FTO in EGFR tyrosine kinase inhibitor (TKI) resistance, specifically to erlotinib, in three NSCLC cell lines harboring either wild-type or mutant EGFR. We also performed immunohistochemistry on lung tumor tissues from patients diagnosed at different stages of NSCLC. **Results**: Our study found an upregulation of FTO in erlotinib-resistant (ER) cell lines at both the gene and protein levels. FTO inhibition and knockdown significantly reduced cell viability of erlotinib-resistant H2170 and PC9 cells by over 30% when treated with 0.8 µM of Dac51 and about 20% when treated with siFTO. FTO inhibition also slowed down the migration of ER cells, and the effect was even more pronounced when combined with erlotinib. Furthermore, FTO was found to be overexpressed in late-stage NSCLC tumor tissues compared to early-stage tumors, and it was upregulated in patients who smoked. **Conclusions**: These findings suggest FTO might mediate resistance and tumor growth by augmenting cell proliferation. In addition, FTO can be a potential prognostic marker in NSCLC patients.

## 1. Introduction

Lung cancer contributes to about 20% of cancer-related mortality and is expected to cause approximately 124,730 deaths in 2025 [1]. It is categorized into two subtypes, namely, non-small cell lung cancer (NSCLC) and small cell lung cancer (SCLC), which have different therapeutic and management options [2]. The NSCLC 5-year survival rate is 25.4%, with a 68% survival rate when diagnosed at the early stage (IB) compared to a 0–10% survival rate at an advanced stage (IVA-IVB) [3,4]. Adenocarcinoma, which originates from alveolar cells lining the small airway epithelium, is the most common NSCLC subtype and accounts for about 40% of all lung cancer diagnoses [5]. Approximately two-thirds of the NSCLC harbors oncogenic mutations, and some of these mutations have become therapeutic targets [6]. Epidermal growth factor receptor (EGFR), one such oncogenic driver, is often found to be amplified and mutated. It has been found to be overexpressed in greater than 60% of NSCLC cases [7]. EGFR can be stimulated by its extracellular ligand, epidermal growth factor (EGF), which dimerizes and activates its tyrosine kinase (TK) domain. This further stimulates downstream signaling cascades such as mitogen-activated protein kinase (MAPK), phosphatidylinositol 3-kinase, and protein kinase B (PI3-PKB/Akt), a signal transducer and an activator of transcription pathways and STAT3 proteins [8]. More than 90% of the cases with EGFR tyrosine kinase mutations show short in frame deletions in exon 19 and point mutations (L858R) in exon 21. These mutations lead to constitutive activation of downstream signaling pathways, leading to cell proliferation and cancer progression [9,10].

Tyrosine kinase inhibitors (TKI) suppress phosphorylation of the tyrosine kinases and prevent downstream signaling [11]. Erlotinib and gefitinib are first-generation EGFR TKIs that have been proven efficacious in treating NSCLC. They cause competitive, reversible binding to adenosine triphosphate binding sites at the tyrosine kinase domain [9]. Though these EGFR TKIs are potent, patients often develop resistance after 9–12 months of treatment due to secondary mutations such as T790M (found in 50% cases with acquired resistance). This renders the first generation of EGFR TKIs ineffective and overcomes the efficacy of second-generation EGFR TKIs, such as afatinib [12]. In addition to T790M, NSCLC patients acquire resistance to EGFR TKIs by upregulating alternative signaling mechanisms, such as PI3K/AKT/mTOR and JAK2/STAT3 pathways, epithelial–mesenchymal transition (EMT) phenotypic transformation, and several other receptor tyrosine kinases (RTKs), such as c-MET, IGF-1 receptor human epidermal HER2, and HER3 [9,12]. Osimertinib, a third generation EGFR TKI, is effective against T790M and has been FDA-approved as a first line treatment for NSCLC patients with EGFR exon 19 deletions, L858R or T790M mutations [13].

The fat mass and obesity-associated gene (FTO), initially identified by Genome-Wide Association Studies (GWAS) as being associated with obesity related traits [14], disease susceptibility, and metabolic processes [15], was later found to be implicated in various cancers [16]. For instance, FTO SNPs have been found to be associated with increased risk for breast cancer in multiple ethnic groups [17,18]. Studies have also found an association between FTO SNPs and melanoma susceptibility [19]. More current studies have suggested FTO could suppress tumor growth, potentiate immunotherapy, and overcome drug resistance [20]. Nevertheless, current studies have not shown a connection between FTO SNPs and FTO expression in cancer patients.

**Figure 1:**
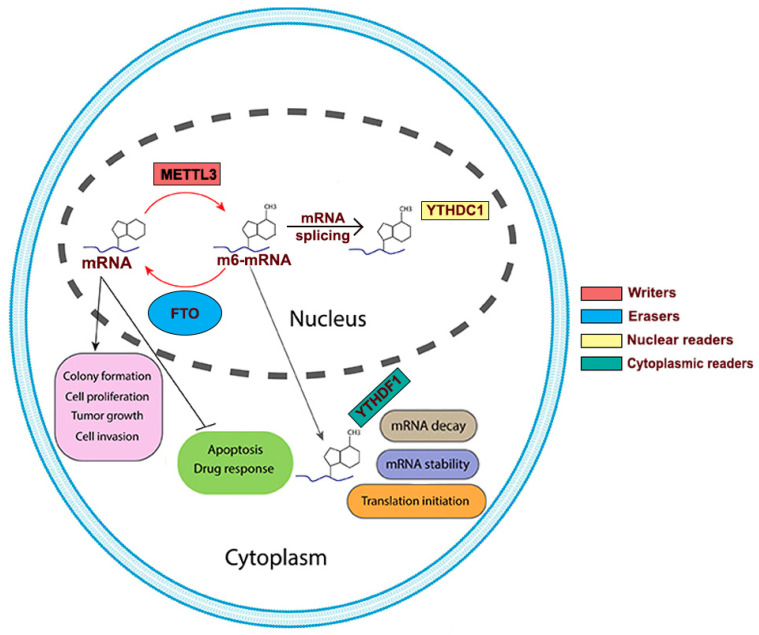
Modification at N6 position of adenosine in mRNA (m6-mRNA) by FTO. Writer proteins such as METTL3 add a methyl group at the N6 position of mRNA, which is recognized by several reader proteins like YTHDC1. This leads to various post-transcriptional effects like mRNA decay, mRNA stability, and translation. However, eraser proteins, such as FTO, remove the N6 methyl group, promoting colony formation, cell proliferation, and tumorigenesis in various cancers. Studies also suggest that removal of this methyl group lowers drug response and apoptosis in cancer.

FTO’s role in cancer was likely due to its demethylation activity directed at N6-methyladenosine (m6A) [21,22]. The m6A is the most common internal transcript modification in eukaryotic RNA, and its interaction with “reader” proteins affects RNA metabolic processes such as RNA splicing, degradation, and translation (Figure 1) [23]. Demethylation of m6A RNA by FTO may regulate the stability of different RNA targets in various cancers [23,24]. Overexpression of FTO can cause downregulation in expression of m6A [24]. In lung cancer, FTO downregulation increases m6A levels which then mediates downregulation of MZF1, a myeloid zinc finger protein 1 [24] and USP7, ubiquitin-specific protease 7 due to decreased stability of their mRNA transcripts, causing an increase in lung cancer tumorigenicity [25]. FTO’s demethylase function and its association with cancer has gained attention in recent years [26]. FTO upregulation that increases demethylation of m6A has been associated with melanoma [27] and causes downregulation of BNIP3, a tumor suppressor gene in breast cancer [28]. FTO is also associated with many other cancers, such as gastric cancer, advanced non-small cell lung cancer (NSCLC), leukemia, and hepatocellular carcinoma [27,29,30]. However, the potential of FTO inhibition and FTO knockdown to overcome erlotinib and osimertinib (3rd generation EGFR TKI) resistance has not been established. The effect of smoking on FTO expression in NSCLC patients has also not been studied. Furthermore, the expression of FTO in both early and late-stage lung cancer, as well as its correlation with patient survival using a Kaplan–Meier curve, has yet to be investigated. By studying the role of FTO in lung tumors and defining mechanisms of overcoming drug resistance through FTO modulation, this study may improve NSCLC patient prognosis.

Additionally, FTO protein may act as an oncogene regulating the phenotypic malignancies of cancer in an m6A-dependent manner and may contribute to therapy resistance [15]. Studies found that FTO enhances resistance to chemoradiotherapy in cervical squamous cell carcinoma [15,31]. FTO overexpression in leukemia can also generate a resistant phenotype against EGFR TKIs [15,32], and its downregulation resensitizes melanoma cells to interferon gamma (IFN-γ) and anti-programmed cell death protein 1 (PD1) [15,27].

Individuals with lung cancer have been observed to show elevated expression of FTO. FTO stabilizes ubiquitin-specific protease 7 (USP7) RNA levels, promoting lung cell growth and increasing tumorigenicity in mice [25]. Overexpression of FTO in lung cancer cells could also enhance myeloid zinc finger protein 1 (MZF1) expression leading to oncogenesis [24]. While several studies have found that FTO upregulation promotes tumor cell proliferation and invasion and suppresses apoptosis, a recent publication demonstrated that widespread FTO downregulation induces invasion and metastasis in epithelial cancers, and FTO knockdown triggers Wnt-mediated epithelial–mesenchymal transition [33,34]. These studies present controversial perspectives on the effect of FTO on cancers and warrant further investigations to validate the role of FTO in lung tumor growth and drug resistance.

In this investigation, we explored the role of FTO in lung cancer tumorigenicity. We found upregulation of FTO expression in EGFR TKI cell lines and that the combinatory effects of FTO inhibitors and EGFR TKIs were additive. The knockdown of FTO increased ATP levels and tumorigenicity, as seen by migratory and wound healing assays. These studies indicate that FTO may play a role in EGFR TKI resistance and that it could be a target for EGFR TKI resensitization. In addition, we discovered that FTO might be a potential prognostic biomarker since its expression is upregulated in late-stage NSCLC and in smokers.

## 2. Materials and Methods

### 2.1. NSCLC Cell Lines and Cell Culture

Four NSCLC cell lines, H2170, H358 (wild-type EGFR), H3255 (L858R EGFR mutation), and H1975 (L858R and T790M EGFR mutations), were cultured following the protocol of the American Type Culture Collection (ATCC). PC9 is a lung adenocarcinoma cell line with EGFR exon 19 deletion. All cell lines were cultured in Roswell Park Memorial Institute (RPMI) 1640 (Thermo Fisher Scientific, Pittsburg, PA, USA) supplemented with 10% fetal bovine serum (FBS, Atlanta Biologicals, Lawrenceville, GA, USA), 1% antibiotic–antimycotic solution (Life Technologies, Carlsbad, CA, USA), 1% sodium pyruvate (1 mM) (Life Technologies, Carlsbad, CA, USA), and 1% 4-(2-Hydroxyethyl) piperazine-1-ethane-sulfonic acid (HEPES, 1 mM) (Life Technologies, Carlsbad, CA, USA). The cells were grown in a humidified incubator at 37 °C with 5% CO_2_.

Cell lines were made resistant to EGFR TKIs osimertinib and erlotinib in a dose-dependent manner for 16 weeks (see Supplementary Table S1 for IC_50_ values) as described earlier [35]. These cell lines have also been used in our previous studies [36,37,38]. H2170 ER, H358 ER, H1975 ER, and PC9 ER were cultured in complete media supplemented with 10 µM, 10 µM, 10 µM, and 13 nM erlotinib, respectively, whereas H2170 OR, H358 OR, H3255 OR, and PC9 OR were cultured in media supplemented with 5 µM, 10 µM, 24 nM, and 21 nM Osimertinib, respectively. All parental cell lines were trypsinized with 0.25% Trypsin (0.53 mM EDTA solution) (Corning Cellgro, Corning, NY, USA), and the erlotinib/osimertinib-resistant (ER/OR) cell lines were treated with 0.05% Trypsin (0.53 mM EDTA) (Corning Cellgro, Corning, NY, USA).

### 2.2. Tyrosine Kinase Inhibitors, Epidermal Growth Factor Ligands, and FTO Inhibitor

Erlotinib hydrochloride from LC Laboratories (Woburn, MA, USA) was reconstituted in DMSO at 20 mM, and osimertinib from Selleck chemicals (Houston, TX, USA, Catalog No. S7297) was reconstituted in DMSO at 10 mM. Epidermal growth factor (EGF) from Peprotech (Rocky Hill, NJ, USA) was dissolved in PBS at 15 ng/µL. Dac51 (S9876), a selective and potent FTO inhibitor, from Selleck chemicals (Houston, TX, USA) was reconstituted in DMSO to a stock concentration of 5 mM. All aliquots of these reagents were stored at −20 °C.

### 2.3. siRNA Transfection

Mock siRNA (Santa Cruz Biotechnology, Dallas, TX, USA, Cat No. sc-37007), FTO-siRNA (Horizon Discovery, Cambridge, UK, Catalog ID: M-004159-01-0005), and Dharmafect-2 (Horizon Discovery, Cambridge, UK, Cat. No. T-2002-01) was diluted in Opti-MEM media (Life Technologies, Carlsbad, CA, USA, Cat No. 31985-070) separately and incubated for 5 min. Diluted siRNA and Dharmafect-2 were then mixed in a 1:1 ratio to reach a 100 nM siRNA final concentration and incubated for 20 min at room temperature. This mixture was then added to individual wells/dishes seeded with cells.

### 2.4. qPCR

Primers (Table 1) from Integrated DNA Technologies (IDT, Coralville, IA, USA) were reconstituted in nuclease-free water to 100 µM and were diluted to a working concentration of 10 µM for the PCR reaction. Glyceraldehyde-3-phosphate dehydrogenase (GAPDH) was used as the endogenous positive control for the reaction process. 1 × 10^6^ cells were plated in 60 mm dishes and were grown to 90% confluency followed by incubation with serum-free media (RPMI + 0.5% BSA) for 24 h. To harvest lysate, 1 mL of Trizol (Sigma Aldrich, St. Louis, MO, USA, Cat. No. T9424) was added to each dish. RNA was isolated using chloroform, isopropanol, and ethanol. 2 µg of RNA was used in each 20 µL RT-PCR reaction for cDNA conversion (Applied Biosystems, Foster City, CA, USA, Cat. No. 00789517). qPCR was conducted with the cDNA products using PowerUp SYBR green from (Applied Biosystems, Vilnius, Lithuania, Cat. No. A25742). Fold changes in gene expression were calculated from 2^(−Δ(ΔCt))^. Each reaction was conducted using technical triplicates, and the experiment was repeated three times.

### 2.5. Immunoblotting

First, 350,000 cells were seeded in 35 mm dishes. When they reached 90% confluency, the cells were incubated in serum-free media (RPMI with 0.5% BSA) for 24 h, followed by treatment with erlotinib in serum-free media for 24 h. Finally, the cells were treated with 15 ng/µL EGF ligand for 2.5 min before lysate collection. Lysates were collected, electrophoresed, and transferred onto nitrocellulose membrane (Bio-Rad, Hercules, CA, USA, Cat. No. 162-0112).

Primary monoclonal antibodies for FTO (rabbit) (Cat. No. MA5-33221) and GAPDH (mouse) (Cat. No. MA5-15738), and secondary antibodies anti-rabbit IgG (Cat. No. 31460) and anti-mouse IgG (Cat. No. A16078) were obtained from Invitrogen (Waltham, MA, USA). All the primary antibodies were diluted to 1:5000 in 1% BSA made in 1× TBST. Secondary antibodies were made at 1:10,000 dilutions with 1% blocking grade milk (Bio-Rad, Hercules, CA, USA, Cat. No. 170-6404XTU).

The protein bands on the membrane were scanned using ChemiDoc Imaging System (Bio-Rad, Hercules, CA, USA), and ImageJ software (version 1.46, National Institutes of Health, Bethesda, MD, USA) was then used for densiometric analysis. Data are represented as the average of two independent experiments.

### 2.6. Immunofluorescence

An 8-well chamber slide (Lab-Tek II Chamber Slide System, ThermoFisher Scientific, Waltham, MA, USA, Cat. No. 154461) was coated with poly-L-lysine (0.01% solution) (Sigma-Aldrich, St. Louis, MO, USA, Cat. No. P4707) and seeded with 8000 cells per well. When the cells reached 80% confluency, they were fixed with 4% paraformaldehyde, permeabilized for 15 min using 0.1% Triton X-100 at RT, and blocked using 5% normal goat serum (Vector Laboratories, Burlingame, CA, USA, Cat. No. S-1000) and 0.2% fish gelatin for 30 min at RT.

The slides were stained with FTO primary antibody (1:250), anti-rabbit IgG secondary antibody (1:250) conjugated with Alexa Fluor 488 (ThermoFisher Scientific, Waltham, MA, USA, Cat. No. A-21206) or Alexa Fluor 555 (ThermoFisher Scientific, Waltham, MA, USA, Cat. No. A-21428), and Hoechst dye [1:2500] (Life Technologies, Carlsbad, CA, USA, Cat. No. 33342). The average intensity of fluorescence of images captured by Olympus Fv10i Fluoview confocal microscope was quantitatively measured using ImageJ software (version 1.46, National Institutes of Health, Bethesda, MD, USA). Data are represented as the average of two independent experiments.

### 2.7. MTT Viability Assay

First, 6000 cells/well were seeded in 96-well plates and allowed to grow to 60% confluency in complete RPMI media. The cells were then treated with different concentrations of Dac51 and/or erlotinib for 48 h. The cells were incubated with 10% MTT reagent prepared in Opti-MEM media for 2 h at 37 °C, followed by treatment with MTT solubilization reagent (acidic isopropanol). Absorbance was measured at 570 nm, and the percentage of cell viability was calculated. Data are represented as the average of two independent experiments. Each experiment had at least technical triplicates for each treatment condition.

### 2.8. Wound Healing Assay

First, 250,000 cells/well were seeded in a 24-well plate and were allowed to grow until 100% confluency in RPMI media. A 200 µL tip was used to create a scratch in the field. The cells were then treated with erlotinib and/or Dac51 for 48 h at 37 °C. Images were taken at 40× magnification at 0-h, 24-h, and 48-h time points and analyzed using T-scratch software (version 1.0, ETH Zurich, Zurich, Switzerland). Data are represented as the average of two independent experiments.

### 2.9. Transwell Migration Assay

First, 30,000 cells/insert (upper chamber) were seeded in 200 μL of incomplete media in a 24-well transwell migration plate. Then, 750 μL of complete media supplemented with 2 ng/mL EGF (chemo-attractant) and different treatments were added to the wells (lower chamber). The plate was incubated for 48 h at 37 °C and 5% CO_2_. The top layer of the membrane in inserts was carefully wiped using a cotton swab to remove the cells that did not migrate through (to avoid false staining). The cells in the inserts were then stained with a Hema-3 stain kit (Fisher Scientific, Waltham, MA, USA, Cat. No. 23-123869) and viewed under the light microscope at 40× magnification. Data are represented as the average of two independent experiments.

### 2.10. ATP Assay

First, 20,000 cells/well were seeded in a 96-well plate and transfected with FTO-siRNA. After 48 h of transfection, the cells were rinsed 2 times with ice-cold PBS. After washing with PBS, the cells were incubated for 30 min in glucose-free media with or without 10 mM oligomycin, a specific inhibitor of ATP synthase to reduce levels of endogenous ATP. Following this, the cells were lysed using the lysis buffer containing phospho-extraction buffer (Boston Bioproducts, Milford, MA, USA, Cat. No. BP-116P) for 5 min on an orbital shaker, after which we provided the luciferase substrate from an ATP determination kit (Invitrogen, Waltham, MA, USA, Cat. No. A22066), followed by an incubation at 37 °C for 5 min. The plates were then incubated in the dark for 10 min, and luminescence was measured using the BioTek Synergy 2 multi-mode microplate reader (BioTek, Winooski, VT, USA). Data are represented as the average of two independent experiments. Each experiment had at least technical triplicates for each condition.

### 2.11. NSCLC Patient Tissues

Formalin-fixed and paraffin-embedded lung tissues were sectioned onto slides. All cases were retrieved from the pathology archives at the OSF St. Anthony’s and UW Health Swedish American Hospital (Rockford, IL, USA) according to an approved Institutional Review Board (IRB) protocol.

### 2.12. Immunohistochemistry

The slides containing tissue sections were hydrated, deparaffinized in xylene, and rehydrated in a series of decreasing concentrations of ethanol (100%, 95%, 70%). Antigen retrieval was carried out using a Tris-EDTA with Tween 20 solution in a 95 °C water bath for 30 min. The slides were blocked with hydrogen peroxide, avidin/biotin blocking kit (Vector laboratories, Burlingame, CA, USA, Cat. No. SP-2001), and 7.5% normal goat serum (Vector laboratories, Burlingame, CA, USA, Cat. No. S-2000-20). The sections were incubated with FTO primary antibody (1:1800) overnight at 4 °C and biotinylated horse anti-rabbit secondary antibody (1:5000) (Vector laboratories, Burlingame, CA, USA, Cat No. BA-2000). Detection was achieved using Vectastain Elite ABC-HRP Kit (Vector laboratories, Burlingame, CA, USA, Cat. No.: PK-6100) followed by an Impact DAB substrate kit (Vector laboratories, Burlingame, CA, USA, Cat. No.: SK-4105). The slides were counterstained with Hematoxylin (Sigma-Aldrich, St. Louis, MO, USA, Cat. No.: MHS 16-500ML), rinsed with tap water, and dehydrated in increasing concentrations of ethanol and xylene.

Images were captured on a KEYENCE BZ Microscope at 40× magnification. The brightness score was correlated with protein expression using HRP and DAB processed brown stained proteins of interest, which were generated by the BZX-800 Analyzer software (version 1.1.8, Keyence, Osaka, Japan).

The H-score was calculated based on a pathologist’s evaluation of tumor slides on a scale of 0–300. The H-score was obtained by grading the intensity of protein expression in the tumors from 0 to 3. This is based on the antibodies’ ability to bind to the protein of interest. The protein expression levels were 1 = weak staining, 2 = moderate staining, and 3 = strong staining. The final score was calculated by multiplying the scores by the percentage of stained cells. The scores ranged from 0–300. Calculation for H-score = 1 (% cells with weak expression) + 2 (% cells with moderate expression) + 3 (% cells with strong expression). A two tailed *t*-test analysis was used to determine the statistical significance.

### 2.13. Statistical Approaches

Statistical analyses were performed using Microsoft Excel (Microsoft 365, version 2505) and GraphPad Prism (version 10.0.0). Data were compared to appropriate control groups, as specified in the figure legends. Comparisons between two groups for continuous variables were conducted using an unpaired two-tailed *t*-test. For comparisons involving more than two groups, a one-way ANOVA with Dunnett’s post hoc test was used. For categorical variables, the Fisher’s exact tests were performed. Kaplan–Meier curves were generated using GraphPad Prism to estimate survival probabilities, and differences were assessed using the log-rank test. A *p*-value < 0.05 was considered statistically significant.

## 3. Results

### 3.1. FTO Upregulation in EGFR-TKI-Resistant Wild-Type EGFR and EGFR-Mutated NSCLC Cells

The mechanisms of lung cancer tumorigenesis are vastly complex, and the relationship between tumor growth and resistance to EGFR TKIs merits investigation. The previous studies [24,25] suggest that FTO might contribute to tumor growth; hence, we performed gene and protein expression analysis on NSCLC cell lines to examine whether FTO is upregulated in EGFR-TKI-resistant cell lines.

**Figure 2:**
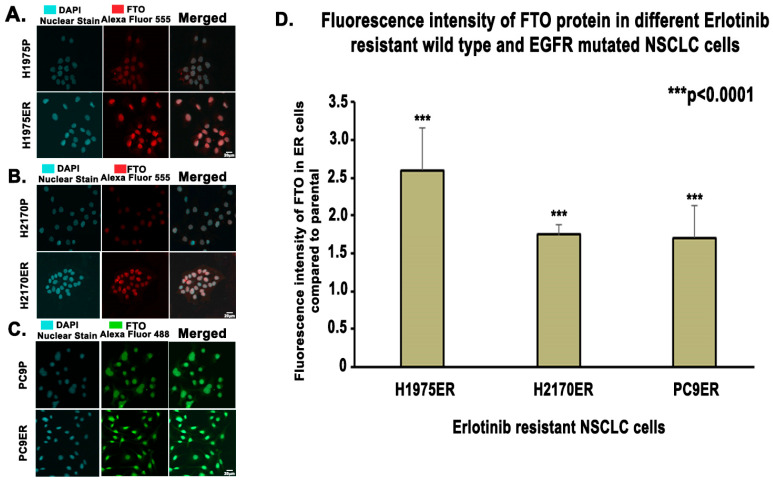
Localization of FTO in EGFR-TKI-Resistant Wild-Type and EGFR-Mutated NSCLC Cells. First, 20,000 cells were plated per well in an 8-well chamber slide then fixed and probed with FTO antibodies. DAPI was used as a nuclear stain; Alexa 488 and Alexa 555 conjugated secondary antibodies were used to detect FTO in PC9 cell line and for H1975 and H2170 cell lines, respectively. Images were captured using Olympus fv10i confocal microscope and localization was analyzed using the ImageJ software (**A**–**C**) Expression of FTO was found mainly in the nucleus, and the expression was significantly higher in the erlotinib-resistant cells (**D**) The average fluorescence intensity was higher in H1975 ER, H2170 ER, and PC9 ER cells by 2.6-fold, 1.8-fold, and 1.7-fold, respectively, compared to the parental cells, and the results were statistically significant by the two-tailed *t*-test analysis.

**Figure 3:**
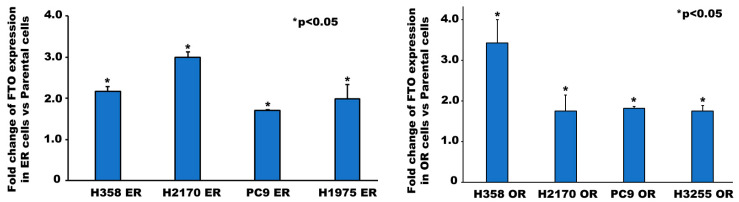
FTO Gene Expression in Wild Type and EGFR-TKI-Resistant NSCLC Cells. First, 3.5 × 10^5^ cells were seeded in a 35 mm dish and allowed to grow for 24 h, following which the cells were starved for 24 h and then processed for total mRNA isolation. The mRNA was quantified and analyzed using qPCR for mRNA levels of genes of interest. FTO gene expression was significantly upregulated in erlotinib-resistant cells (H358 ER, H2170 ER, PC9 ER, and H1975 ER) and osimertinib-resistant cells (H358 OR, H2170 OR, PC9 OR, and H3255 OR) compared to their parental cells using two tailed *t*-test. The results from qPCR were normalized with GAPDH.

**Figure 4:**
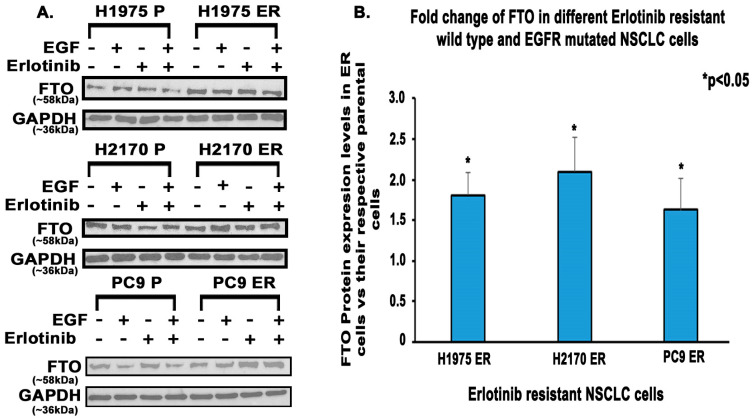
FTO Protein Modulation in the Erlotinib-Resistant Wild-Type and EGFR-Mutated NSCLC Cell Lines. (**A**) First, 3.5 × 10^5^ cells were seeded in a 35 mm dish and allowed to grow for 24–48 h until reaching 90% confluency, after which the cells were treated with serum free media (Plain media with 0.5% BSA) for 24 h, and then the cells were either treated with EGF (15 ng/mL for 2.5 min) and/or 10 µM erlotinib (1975 ER and 2170 ER) and 13 nM (PC9 ER) for 24 h. (**B**) The immunoblotting results showed that the FTO protein was 1.8-fold, 2.1-fold, and 1.6-fold higher in H1975 ER, H2170 ER, and PC9 ER cell lines respectively. The fold change in parental cells was considered 1-fold. The modulations in the protein expression were calculated by densitometric analysis using the ImageJ software (version 1.46, National Institutes of Health, Bethesda, MD, USA), and the results were statistically significant by the two-tailed *t*-test analysis.

FTO was upregulated at gene and protein levels in multiple EGFR-TKI-resistant NSCLC cell lines. These cell lines were made resistant to EGFR TKIs by continuously exposing them to IC_50_ and higher concentrations of EGFR TKIs (Supplementary Table S1) [35,36,37,39]. Immunofluorescence studies demonstrated a 1.8-fold, 1.7-fold, and 2.6-fold increase in FTO expression in erlotinib-resistant (ER) H2170, PC9, and H1975 cells compared to their parental cell lines, respectively (Figure 2). FTO was found localized in the nucleus of both ER and parental cells (Figure 2A–C). RNA levels were significantly elevated in both erlotinib- and osimertinib-resistant cells (Figure 3). Furthermore, all three ER cell lines demonstrated an increase in FTO protein expression relative to their parental cell lines even in the presence of both EGF and erlotinib (Figure 4A). FTO protein was 1.8-fold, 2.1-fold, and 1.6-fold higher in H1975 ER, H2170 ER, and PC9 ER cells, respectively, compared to their parental cells (Figure 4B).

### 3.2. FTO Protein Modulation in the Erlotinib-Resistant Wild Type and EGFR-Mutated NSCLC Cells

FTO upregulation in EGFR TKI lung cancer cells indicates that FTO could promote tumorigenesis of resistant cells. To elucidate how FTO supports tumor growth, we determined whether FTO inhibition or knockdown influences cell proliferation and cell migration/expansion.

**Figure 5:**
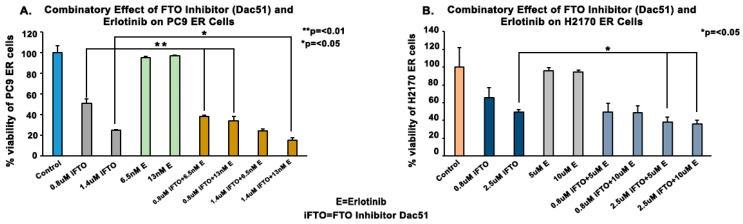
Effect of FTO Inhibition on the Efficacy of Erlotinib in EGFR-TKI-Resistant PC9 and H2170 ER cells. First, 6000 ER cells per well were plated in a 96-well plate. Once the required confluency was achieved, the lanes were treated with varying concentrations of Dac51 and erlotinib to study the efficacy of the combination. (**A**,**B**) We found the Dac51 and erlotinib had a more than additive effect in PC9 ER (0.8 µM Dac51 with 13 nM erlotinib) and H2170 ER (2.5 µM Dac51 with 10 µM erlotinib). The drug and inhibitor combination displayed a 13.4% and 7.7% increase in inhibition compared to the drug and inhibitor individually in PC9 ER and H2170 ER cells, respectively. Thus, the effectiveness of the drug seems to be enhanced by the FTO inhibitor, hence, suggesting FTO’s potential to combat EGFR TKI-Resistance. These results were found to be significant using the one-way ANOVA with Dunnett’s post hoc test.

**Figure 6:**
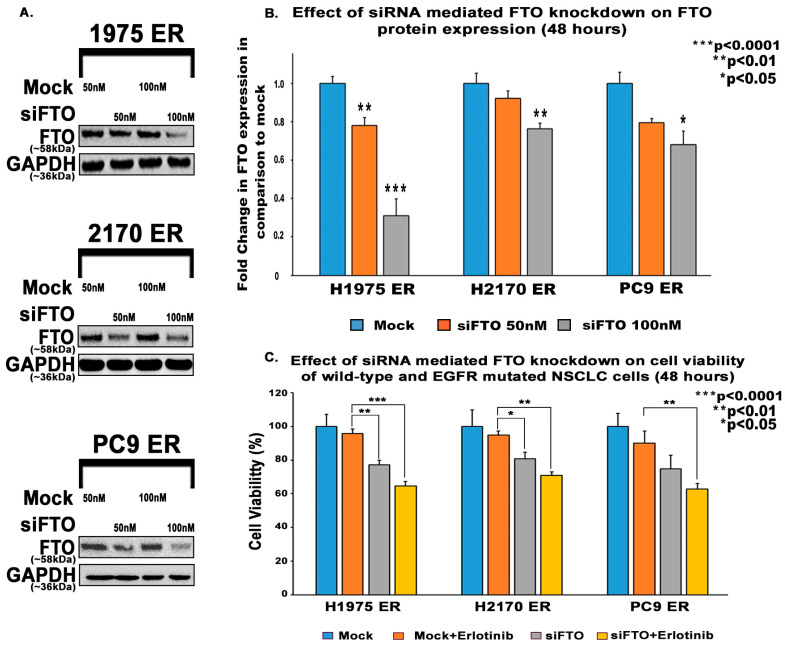
The effect of FTO knockdown on cell survival in erlotinib-resistant NSCLC cells. (**A**) First, 5500 cells/well were seeded in a 96-well plate in antibiotic-free media a day before transfection. The cells were then transfected with either mock or FTO siRNA using Dharmafect-2 for 24 h. Cells were then treated with erlotinib (10 µM for H1975 ER and H2170 ER, 13 nM for PC9 ER cells) for 48 h. (**B**) Downregulation of FTO on H1975 ER, H2170 ER and PC9 ER cells after siRNA treatment. (**C**) The percentage cell viability was calculated with respect to the viability of mock siRNA plus erlotinib and the results were found to be statistically significant by one-way ANOVA with Dunnett’s post hoc test.

Erlotinib-resistant NSCLC cells were treated with varying concentrations of Dac51 (a specific FTO inhibitor), erlotinib, or a combination of both. The FTO inhibitor reduced cell viability of H2170 ER by 34% and 51% at 0.8 μM and 2.5 μM, and in PC9 ER cells by 54% and 74% at 0.8 μM and 1.4 μM, respectively. The combination effect of erlotinib and FTO inhibitor was close to additive (Figure 5). We then used siRNA to knock down FTO in erlotinib-resistant NSCLC cell lines. At 100 nM of siFTO, the FTO protein was downregulated by 3.2-fold in H1975 ER, 1.3-fold in H2170 ER, and 1.5-fold in PC9 ER cells after 48 h of transfection (Figure 6A). siFTO decreased cell viability significantly in all three erlotinib-resistant cell lines, as cell viability decreased by about 19% to 23% compared to the mock siRNA (Figure 6C).

Nevertheless, the effect of siFTO in addition to erlotinib was additive (Figure 6C). FTO inhibition and knockdown diminished cell survival, implying that FTO is important for cell proliferation. The additive effect of the combination treatment also suggests that FTO stimulates cell proliferation via a pathway different from erlotinib on EGFR.

**Figure 7:**
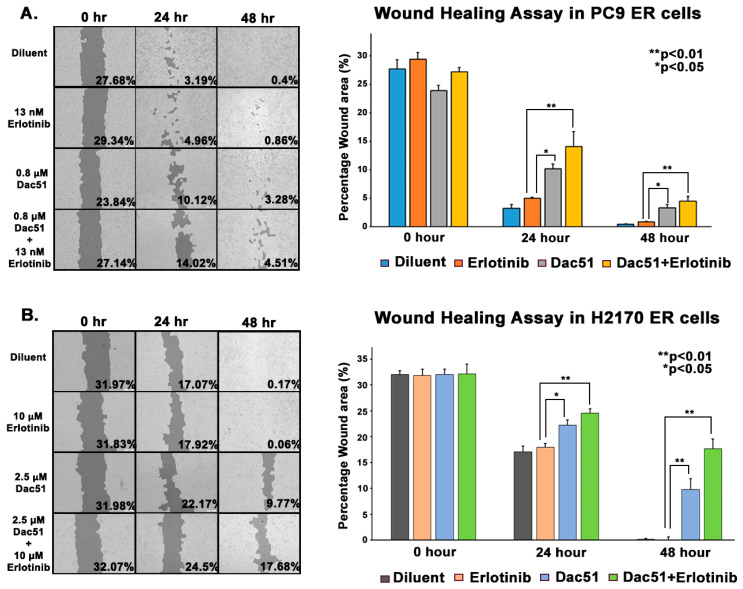
Effect of FTO Inhibition and Erlotinib Treatment on Wound Healing Assay in PC9 and H2170 ER NSCLC Cell Lines. First, 250,000 cells were seeded in a 24-well plate in quadruplets a day before treatment. Once achieving confluency, a scratch was created using a 200 µL tip. The cells were then treated with no treatment condition (diluent), erlotinib, Dac51, and erlotinib + Dac51 for 48 h. The images were captured at the 0-h, 24-h, and 48-h time points after the treatment. The percentage wound area was plotted for all the four conditions, (**A**) Open wound area for PC9 ER cells was 4.51% (calculated from the ImageJ software version 1.46, National Institutes of Health, Bethesda, MD, USA), which was greater than the additive effect of Dac51 and erlotinib individual conditions, whereas (**B**) open wound area for H2170 ER showed to be 17.68%, which was greater than the additive effect of Dac51 and erlotinib individual conditions. The results were found to be significant using one-way ANOVA with Dunnett’s post hoc test.

**Figure 8:**
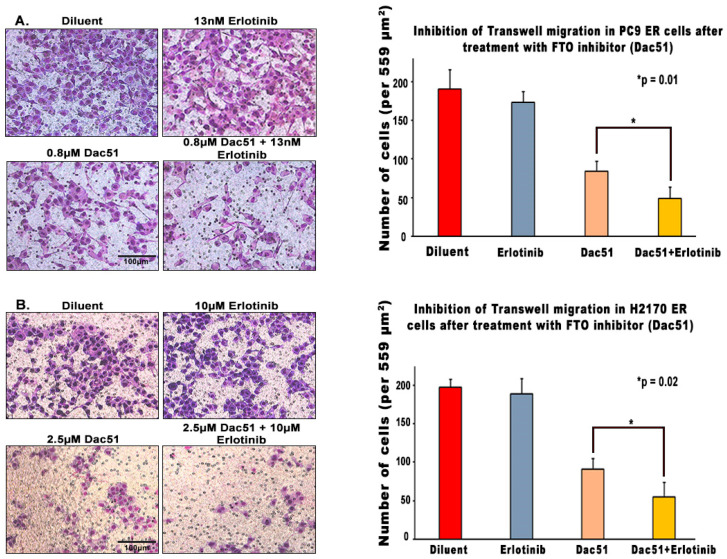
Effect of FTO Inhibition on Transwell Migration in PC9 and H2170 ER NSCLC Cell Lines. First, 30,000 cells/inserts were seeded on the upper membrane of the transwell migration plate insert in incomplete RPMI media containing Dac51, erlotinib, and Dac51 + erlotinib. The lower chamber of the well was given complete RPMI media having FBS and 2 ng/mL EGF, and then the plate was kept for incubation at 37 °C in 5% CO2 for 48 h. After 48 h, the plate was processed using Hema-3 stain kit, and we found (**A**) about 72% and 40% less cells in the combination compared to the erlotinib alone and inhibitor alone, respectively, for PC9 ER and (**B**) about 71% and 40% less cells were found in the combination compared to the erlotinib alone and inhibitor alone, respectively, for H2170 ER cells. The percentage decrease in migration was calculated with reference to the erlotinib alone condition, and the results were found to be statistically significant by two tailed *t*-test analysis.

We then explored whether decreasing FTO levels alters the migratory capacity of lung tumor cells using wound healing assays and transwell assays. Combination treatment slowed down the cell migration more than the FTO inhibitor alone or erlotinib alone in erlotinib-resistant cell lines. After creating a scratch occupying about 27% of the field in PC9 ER cells, Dac51 and combination treatment slowed down wound closure by 11.2% and 14.1%, accordingly (Figure 7A). Similarly, forty-eight hours after creating a scratch occupying about 32% of the field, the wounds of the control and erlotinib-treated wells had closed, whereas Dac51 treatment slowed down wound closure by 30.4% and the combination treatment by 55.1% in H2170 ER cells (Figure 7B). Furthermore, transwell assays demonstrated that combination treatment drastically decreased the number of migrated H2170 ER and PC9 ER cells in both cell lines. Dac51 decreased the number of migrated cells by about 55% compared to diluent, and erlotinib alone reduced that by 4–8%. Combination treatment, on the other hand, lowered the number of migrated cells by 71% (Figure 8A,B). The reduction in both wound healing capacity and number of migrated cells was more than the cumulative effect of erlotinib and FTO inhibitor alone, which indicated that FTO might enhance ER cells’ abilities to migrate and expand.

**Figure 9:**
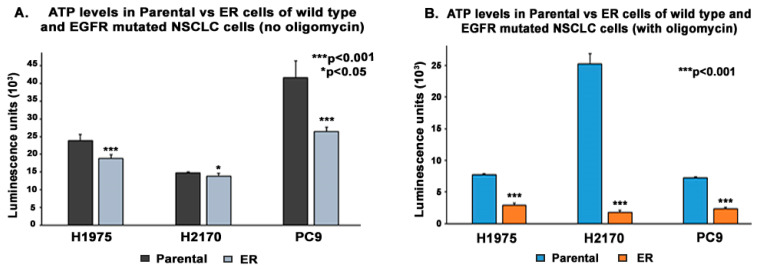
Effect of FTO Dysregulation on Adenosine Triphosphate (ATP) Levels in Wild Type and EGFR Mutated NSCLC Cell Lines. Cells were plated at a cell density of 2 × 10^4^ cells per well in 96-well plates and incubated for 48 h. Following that, cells were rinsed once with RPMI media lacking glucose and sodium pyruvate and incubated for 30 min in either RPMI media lacking glucose and sodium pyruvate or 10 μM oligomycin in RPMI media lacking glucose and sodium pyruvate. Cells were then rinsed twice with HBSS. Cells were then lysed using the mammalian lysis buffer, and ATP concentration was determined by adding the provided substrate and measuring the ATP-associated luminescence using the Biotek Synergy 2 multi-mode microplate reader. (**A**) ATP was downregulated by 1.3-fold in H1975 ER, 1.1-fold in H2170 ER, and 1.6-fold in PC9 ER (without oligomycin) and (**B**) 2.7-fold in H1975 ER, 14.4-fold in H2170 ER, and 3.1-fold in PC9 ER cells (with oligomycin) compared to the respective parental cells. The results were found to be statistically significant by two tailed *t*-test analysis.

We then explored whether FTO levels alter the ATP levels of lung tumor cells using an ATP assay. We found that FTO levels were inversely proportional to the levels of ATP. The cells were cultured in 96-well plates and were provided with glucose free media with or without oligomycin (a specific inhibitor of oxidative phosphorylation) for 30 min before quantifying ATP levels. We found that ATP was downregulated by 1.3-fold in H1975 ER, 1.1-fold in H2170 ER, and 1.6-fold in PC9 ER with no oligomycin treatment (Figure 9A) and 2.7-fold in H1975 ER, 14.4-fold in H2170 ER, and 3.1-fold in PC9 ER cells when treated with oligomycin (Figure 9B) compared to the respective parental cells. The fold change was calculated using luminescence readout in ER cells against their respective parental cells, and the results were found to be statistically significant by two tailed *t*-test analysis.

**Figure 10:**
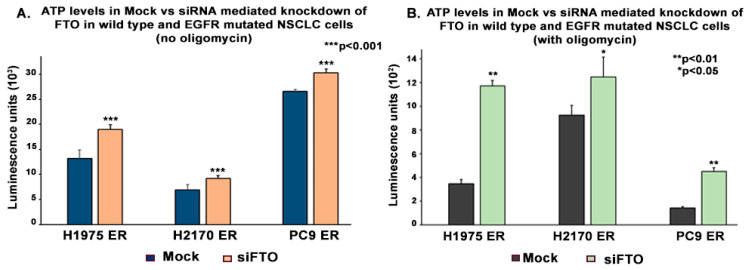
Effect of siRNA-Mediated FTO Knockdown on ATP Levels in Erlotinib-Resistant Wild-Type and EGFR-Mutated NSCLC Cell Lines. Cells were plated at a cell density of 2 × 10^4^ cells per well in 96-well plates for 24 h. Cells were then transfected with 100 nM siFTO or non-targeting (mock) siRNA and then incubated for a total of 48 h post-transfection. Following that, cells were rinsed 1× with RPMI media lacking glucose and sodium pyruvate and incubated for 30 min in either RPMI media lacking glucose and sodium pyruvate or 10 μM oligomycin in RPMI media lacking glucose and sodium pyruvate. Cells were then rinsed twice with HBSS. Cells were then lysed using the mammalian lysis buffer, and ATP concentration was determined by adding the provided substrate and measuring the ATP-associated luminescence using the Biotek Synergy 2 multi-mode microplate reader. (**A**) ATP was upregulated by 1.4-fold in H1975 ER, 1.3-fold in H2170 ER, and 1.2-fold in PC9 ER (without oligomycin) and (**B**) 3.4-fold in H1975 ER, 1.3-fold in H2170 ER, and 3.2-fold in PC9 ER cells (with oligomycin) compared to the respective mock siRNA-transfected cells The results were found to be statistically significant by two tailed *t*-test analysis.

To further confirm the role of FTO in the ATP modulation, we downregulated FTO expression using siRNA. For this study, we performed an ATP assay on H1975 ER, H2170 ER, and PC9 ER, after they were transfected with mock siRNA and FTO-specific siRNA. The cells were cultured in 96-well plates and were provided with glucose free media with or without oligomycin for 30 min before quantifying ATP levels. The trend we found earlier was consistent here, as the knockdown of FTO caused ATP levels to increase in the presence and absence of oligomycin. We found that ATP was upregulated by 1.4-fold in H1975 ER, 1.3-fold in H2170 ER, and 1.2-fold in PC9 ER with no oligomycin treatment (Figure 10A) and 3.4-fold in H1975 ER, 1.3-fold in H2170 ER, and 3.2-fold in PC9 ER cells when treated with oligomycin (Figure 10B) compared to their respective mock siRNA transfected cells. The fold change was calculated using the luminescence readout in ER cells against their respective parental cells, and the results were found to be statistically significant by two tailed *t*-test analysis.

### 3.3. FTO Is Highly Expressed in Late-Stage Lung Tumor Tissues

**Figure 11:**
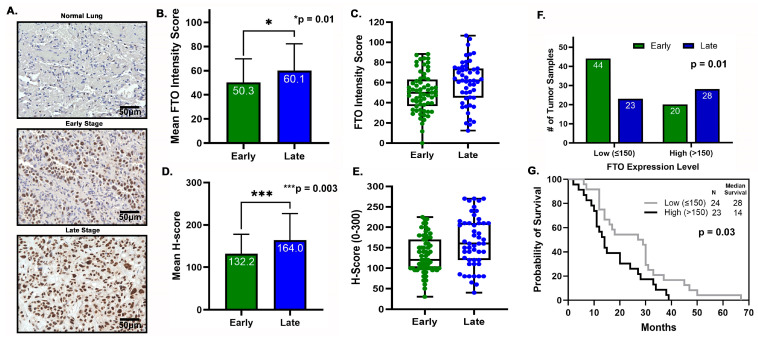
FTO Expression in Early- and Late-Stage Lung Tumor Tissues by Immunohistochemistry. (**A**) Representative images of normal lung tissue, early-stage NSCLC (stages I–IIIA), and late-stage NSCLC (stages IIIB–IV) tumor sections stained for FTO (brown). Images were captured at 40× magnification. (**B**) Imaging analysis was performed on 64 early-stage and 51 late-stage lung tumor samples. The cumulative mean FTO intensity scores for early- and late-stage tumors were plotted. Statistical significance was determined using a two-tailed t-test. (**C**) Box plot showing individual mean intensity scores from the imaging study. Each dot represents the average FTO intensity score from three regions of a given tumor sample. (**D**) H-scores were calculated based on the distribution of weak, moderate, and strong FTO expression, as evaluated by a pathologist. Cumulative mean H-scores are shown for early- and late-stage tumor samples. (**E**) Box plot analysis showing distribution of H-score in early- and late-stage NSCLC cases. (**F**) FTO expression was further classified into low and high expression groups, using an H-score cut-off of 150 (the midpoint of the 0–300 H-score range). Statistical significance was assessed using Fisher’s exact test. (**G**) Kaplan–Meier survival analysis of 47 NSCLC patients comparing overall survival between those with high and low FTO expression.

To study whether FTO participates in tumor progression of non-small cell lung cancers and exhibits prognostic potential, we studied FTO expression in lung tissues of patients diagnosed at different tumor stages (Supplementary Table S2). Of the 115 tumor sections, 85% were adenocarcinomas. Sixty-four were early-stage NSCLC tumors (I-IIIA), and 51 (IIIB-IV) were at advanced, late stages. Both early- and late-stage NSCLC samples expressed FTO predominantly localized to the nuclei (Figure 11A). Late-stage lung cancer tissues had statistically significantly (*p* = 0.01) higher FTO expression compared to early-stage tissues by both imaging study and pathology grading (Figure 11B,D). We also created a dot plot to quantify the FTO intensity and H-scores from patients with late-stage tumors compared to early-stage tumors (Figure 11C,E). When dividing patients into low and high expression levels, we found that 68.7% of early-stage tissue samples exhibited low expression level, and 55% of late-stage tumor tissues had high FTO expression (Figure 11F). Additionally, high expression of FTO was associated with late-stage lung cancer as seen by Fisher’s exact test (*p* = 0.01). With the available survival data, 47 cases including both early- and late-stage NSCLC were analyzed. Patients who have tumors with low FTO expression lived 14 months longer than those with high FTO level (Figure 11G). Hence, high FTO expression is associated with poorer survival (*p* = 0.03). These findings suggest that FTO could be a potential marker for poorer prognosis and warrant studies to explore the role of FTO in promoting tumor growth.

### 3.4. FTO Is Highly Expressed in Patients That Smoke

**Figure 12:**
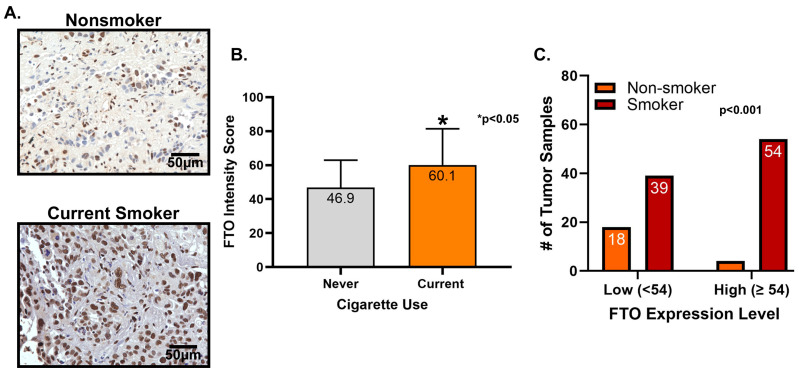
Expression of FTO in Lung Cancer Tissues from Smokers and Non-Smokers. (**A**) NSCLC tumor sections from smokers and non-smokers stained for FTO (brown), with NSCLC smoker patients showing higher expression of FTO relative to NSCLC nonsmoker patients. FTO expression was mainly localized to the nucleus. Images were taken at 40× magnification. (**B**) Graphical representation of FTO intensity using a FTO intensity score obtained using the BZ-X810 Keyence microscope in 22 non-smokers and 28 smokers. The results indicated that patients who smoked had a higher expression of FTO when compared to their non-smoker counterparts. The results were found to be statistically significant using *t*-test. (**C**) Graphical representation of high and low expressions of FTO in the 22 non-smoker and 93 NSCLC smoker patients.

The relationship that exists between cigarette smoking and the development of lung cancer has been well studied for many years [40]. To further our understanding of FTO expression in NSCLC patients, we aimed to identify if a correlation exists between the use of cigarettes and FTO expression. A total of 50 NSCLC tumor sections were stained (Figure 12A) (28 smokers and 22 non-smokers, Supplementary Table S2). The results indicated that patients who smoked had a higher expression of FTO when compared to their non-smoking counterparts. Using a BZ-X810 Keyence microscope, the intensity of the FTO expression was quantified and determined to be 28% higher in smokers, compared to non-smokers (*p* < 0.05) (Figure 12B). A Fisher’s exact test was used to compare high and low expression of FTO with smoking status for smokers and non-smokers. The correlation between the intensity of FTO in smokers versus non-smokers was found to be statistically significant after dividing the patients into high and low expression groups (Figure 12C). We found smokers had a higher FTO expression when compared to their non-smoker counterparts: 58.1% of smokers’ tumor samples showed high FTO expression compared to 18.1% in non-smokers. The results indicated smokers were 3.2 times more likely to have a higher FTO expression when compared to those that were non-smokers (*p* = 0.001, using Fisher’s exact test). Since smokers are more likely to have a high expression of FTO, this suggests these patients might be more prone to drug resistance and tumor growth.

## 4. Discussion

Tyrosine kinase inhibitors (TKIs) are common therapies for non-small cell lung cancers (NSCLC) harboring specific EGFR-activating mutations. Although many generations of these molecularly targeted inhibitors are available, their clinical uses are substantially limited by acquired resistance [41]. Our study aimed to establish an association between FTO expression and EGFR TKI resistance. Our primary goal was to identify a potential link between FTO and NSCLC tumorigenicity. To explore this, we have also modulated FTO expression using siRNA to observe the effects of downregulation in expression levels of FTO on EGFR TKI-resistance. Our earlier studies with FTO knockdown using neuroblastoma cells indicated that FTO downregulation altered metabolic functions through activation of downstream metabolic mediators including AMPk [42]. Therefore, investigating factors that facilitate EGFR TKI resistance could be a meaningful step toward preventing resistance to these inhibitors. We studied the role of the FTO in EGFR TKI resistance at the gene and protein level in erlotinib-resistant and osimertinib-resistant cells and compared them to parental cell lines. Firstly, immunofluorescence studies were conducted to determine the localization of the FTO gene in the nucleus of ER and parental cells (Figure 2A–C) and its upregulation in ER cell lines (Figure 2D). RNA and protein levels of FTO were then quantified via qPCR and immunoblotting in ER and OR cells that showed increased FTO gene and protein expression compared to parental (Figure 3 and Figure 4). Next, we studied the use of an FTO inhibitor, Dac51, in combination with erlotinib and a nearly additive reduction in cell viability was observed (Figure 5), indicating potential for combination therapy. A similar reduction in viability was also observed when FTO was knocked down via siRNA (Figure 6). Wound healing and transwell migration assays showed reduced wound healing and migratory properties in ER cells, especially in combination therapies (Figure 7 and Figure 8), indicating that FTO may play an important role in altering the migratory capacity of lung tumor cells. Additionally, an inverse relationship was observed between FTO expression and ATP levels (Figure 10). Moreover, IHC studies on early- and late-stage lung tumors shed light on FTO’s role in lung cancer tumorigenicity and its role as a prognostic marker (Figure 11). Finally, we observed the increased expression of FTO in smokers compared to non-smokers (Figure 12).

FTO upregulation was observed in gefitinib-resistant NSCLC cell lines and in vivo models. Meclofenamic acid, a selective FTO inhibitor, has been used to overcome gefitinib resistance in NSCLC cell lines [43,44]. However, FTO’s function in erlotinib- and osimertinib-resistant NSCLC cell lines is not well elucidated. Erlotinib is a first-generation EGFR TKI and is used as a first line treatment for NSCLC patients harboring EGFR-activating mutations. When secondary mutations (such as T790M) arise [45], patients can be treated with osimertinib, a third generation EGFR TKI. Thus, we investigated the role of FTO in erlotinib and osimertinib resistance in EGFR-mutated and wild-type NSCLC cells, and we hypothesized that modulating FTO could be key to overcoming EGFR-TKI resistance in these resistant cell lines. FTO modulation not only enhances proliferation in EGFR-mutated NSCLC cell lines but also in non-EGFR mutated lines [22,46]. This indicates that the role of FTO may not be restricted to NSCLC patients with EGFR mutations but may also be applicable to patients with wild-type EGFR. This may be due to the fact that FTO’s effects may be mediated through alternative oncogenic pathways, including upregulation of UB7 mRNA expression [25,46], modulation of MYC signaling, and activation of FAK pathways [20,46]. These findings imply that FTO modulation may have broader therapeutic implications in NSCLC, extending beyond EGFR-mutated tumors.

We performed qPCR and immunoblotting experiments to check for expression of FTO in NSCLC cell lines. At the gene level, FTO was upregulated in H2170 OR, PC9 OR, H2170 ER, PC9 ER, and H1975 ER cells compared to their parental cells (Figure 3). Similarly, Western blot demonstrated that FTO protein was upregulated in H1975 ER, PC9 ER, and H2170 ER cells (Figure 4). These findings were consistent with previous studies showing FTO enrichment in serum samples and tumor tissues of gefitinib-resistant NSCLC patients [47]. Hence, high expression of FTO at the gene and protein level in EGFR-TKI-resistant cell lines strongly suggests that FTO plays a role in the development of EGFR TKI resistance in non-small cell lung cancer cells.

FTO acts as a demethylase that erases the Methyladenosine from the m6A position of mRNA molecules. Most studies have suggested that FTO is found mainly in the nucleus, but some studies have also indicated the cytoplasmic presence of this protein alongside nuclear expression [48,49,50]. Hence, we examined where FTO is localized in EGFR-TKI-resistant cells compared to their parental cell lines. Immunofluorescence demonstrated that FTO was mainly localized in the nucleus of both erlotinib-resistant and parental cells. We found FTO was also upregulated almost two-fold in erlotinib-resistant cell lines (Figure 2). We confirmed that an excess of nuclear expression of FTO in EGFR-TKI-resistant NSCLC cells is associated with the development of the EGFR TKI resistance in lung cancer cells.

Since FTO overexpression was noted in EGFR-TKI-resistant cell lines, we then investigated whether modulating FTO expression or inhibiting it could overcome erlotinib resistance in resistant cell lines. We used Dac51, a selective FTO inhibitor [51], and siFTO transfection to study the effect of FTO inhibition and knockdown on erlotinib efficacy. Dac 51 was used as it has a low IC50 in vitro and showed an inhibitory effect. Dac51 has also been shown to dampen the glycolytic capacity of tumor cells and has promising results in other studies in inhibiting tumor growth [51,52,53]. We found that the inhibition of FTO in the presence of erlotinib treatment decreased cell viability in comparison to FTO inhibition alone or erlotinib treatment alone (Figure 5 and Figure 6). Although the effect was additive, FTO inhibition might be useful for reducing erlotinib dosing and slowing down resistance because FTO inhibition acts on different pathway(s).

Since FTO increases cell migration and invasiveness, we wanted to see how FTO affects the migratory properties of erlotinib-resistant NSCLC cells through wound healing and transwell migration assays. A previous study revealed that FTO downregulation resulted in suppression of lung cancer cell proliferation and colony formation [25]. Our experiments confirm that FTO downregulation impeded erlotinib-resistant NSCLC cell migration, and combination with erlotinib can efficiently curtail cell migration (Figure 8). A combination treatment with both an FTO inhibitor and EGFR TKI could amplify the inhibitory cell migration response of either drug alone.

Previous reports have also demonstrated that FTO was abundantly expressed in the lungs, and its expression patterns differed in the tumor region compared to the surrounding normal tissue region. To our best knowledge, there has not yet been a study showing differences in FTO expression with respect to the stages of lung cancer. Our immunohistochemistry study found significantly higher FTO expression in the nucleus of the late-stage tumor tissues compared to the early-stage tumor tissues (Figure 11). FTO expression might have a significant prognostic value to differentiate the early stage from more advanced stages of lung cancer. A Kaplan–Meier survival analysis of NSCLC tumor tissues showed that those with low FTO expression had a longer median survival time than those with higher expression (Figure 11). This result agreed with a gene enrichment analysis (GSEA) study that showed that patients with high expression levels of m6A erasers, such as FTO, have poorer survival [54]. FTO expression was also found to be higher in smokers, which, to our knowledge, has minimal earlier studies (Figure 12). Most research regarding the role of FTO in cancer progression has emerged over the past decade. As a result, FTO’s role in cancer progression is continually being elucidated in various types of cancers and cell types. While some studies indicate that inhibiting FTO may accelerate epithelial cancer progression, others present conflicting findings, suggesting the opposite effect. FTO’s role in cancer is complex and highly context dependent. FTO downregulation has been associated with increased invasion and metastasis in various epithelial cancers, including breast and prostate cancers. FTO downregulation activates the Wnt signaling pathway, inducing epithelial-to-mesenchymal transition (EMT) and promoting tumorigenesis [34].

However, our current study revealed a pattern, where FTO upregulation promotes lung cancer growth and migration. Several studies have also supported FTO’s oncogenic role in lung cancer [22,24,46]. For example, FTO upregulation has been shown to drive tumor growth via MZF1 in squamous cell lung carcinoma [24]. In NSCLC, FTO can mediate tumor progression through FTO-mediated autophagy and FAP/integrin/FAK signaling [22,46]. These findings indicate that FTO’s function may vary across different epithelial cancer types and subtypes. FTO acts on the epigenetic level and thus can modulate distinct gene targets involving different molecular signaling pathways. Its activity appears to be highly tissue specific; while EMT may drive tumorigenesis in some epithelial cancers, alternative pathways may predominate in others. The intricate interplay between tumor-suppressive and tumor-promoting mechanisms underscores the complexity of FTO’s involvement in cancer progression.

Our study could be expanded by examining the mechanisms by which FTO affects tumor growth, such as FTO’s effect on cell metabolism and energy balance in tumorigenesis. Additionally, overexpression of FTO in EGFR TKI resistance cells could be further investigated. Similarly, the increased expression of FTO in smokers compared to non-smokers along with poor survival rate warrants further research into the specific cell signaling pathways modulated by FTO in NSCLC. Such studies could also elucidate FTO’s role as a potential prognostic marker by examining a larger cohort and correlating it with survival data and clinical outcomes. Additionally, more in-depth analysis of metabolic pathways altered in osimertinib- and erlotinib-resistant cell lines could shed light on how NSCLC becomes resistant to first and third generation EGFR TKI. Further research regarding NSCLC patients without EGFR mutations could also be beneficial to understand better the role of FTO.

## 5. Conclusions

In this study, we demonstrated that FTO played an important role in tumor proliferation and cell migration, as well as mediating EGFR TKI resistance in non-small cell lung cancer. FTO was overexpressed in late-stage (IIIB-IV) tumors compared to early-stage (IA-IIIA) and could potentially be used as a prognostic marker for NSCLC. We investigated the FTO gene expression using qPCR and found its overexpression in several erlotinib- and osimertinib-resistant cells compared to their respective parental cells. To determine FTO protein expression and its localization in various erlotinib-resistant NSCLC cell lines, we performed Western blotting and immunofluorescence. In addition to gene expression, FTO protein was upregulated in erlotinib-resistant cells compared to their parental cell lines. We determined, through MTT cell viability, transwell migration, and wound healing assays, that inhibition/knockdown of FTO can increase the efficacy of erlotinib in the erlotinib-resistant cells. In our study, we also found FTO to be a potential prognostic marker for lung cancer patients, as the immunohistochemistry studies showed a significant correlation between FTO expression and the stage of lung cancer. We also found the correlation between FTO expression and smoking in patients, in addition to its association with patient’s survival. From our study, we conclude that FTO likely mediates EGFR TKI resistance by enhancing cell proliferation and expansion of resistant cells. Since FTO mediates its effects through a different pathway other than erlotinib, FTO inhibition could be useful for reducing erlotinib dosing and thus slowing down the development of resistance to erlotinib.

## Figures and Tables

**Table 1:** Sequences of qPCR Primers.

Gene	Sequence	Melting Point	DNA Bases
FTO	F: GAATTCTATCAGCAGTGGCAGCTG	58 °C	24
	R: AGCCATGCTTGTGCAGTGTG	58.9 °C	20
GAPDH	F: ATGACATCAAGAAGGTGGTG	52.4 °C	20
	R: CAGGAAATGAGCTTGACAAA	50.9 °C	20

## Data Availability

Data is contained within the article or supplementary material. The original contributions presented in this study are included in the article/supplementary material. Further inquiries can be directed to the corresponding author(s).

## Supplementary Materials

The following supporting information can be downloaded at: https://www.mdpi.com/article/10.3390/biomedicines13071653/s1, Table S1: IC_50_ of Erlotinib and Osimertinib of the NSCLC Cell Lines; Table S2: Demographics of Lung Tissue Samples.
