# Supplementary material for: The Role of Fat Mass and Obesity-Associated (FTO) Gene in Non-Small Cell Lung Cancer Tumorigenicity and EGFR Tyrosine Kinase Inhibitor Resistance
Source: Biomedicines. 2025 Jul 7;13(7):1653. doi: 10.3390/biomedicines13071653 (PMC12292738; doi:10.3390/biomedicines13071653)
Supplement: Supplementary file 1 [file biomedicines-13-01653-s001.zip › biomedicines-3448442-supplementary.pdf]

Supplementary Materials

TABLE S1. IC_{50} OF ERLOTINIB AND OSIMERTINIB FOR NSCLC CELL LINES

Cell Line	EGFR Mutation Status	IC_{50} Erlotinib	IC_{50} Osimertinib
H2170 P	Wild-Type	0.5μM	1μM
H2170 ER		11μM	
H2170 OR			5μM
H1975 P	L858R and T790M	2.5μM	
H1975 ER		25μM	
PC9 P	Deletion in Exon 19	7.5nM	15nM
PC9 ER		30nM	
PC9 OR			25nM
H358 P	Wild-Type	1μM	1μM
H358 ER		11μM	
H358 OR			6μM
H3255 P	L858R		10nM
H3255 OR			26nM

Table S1. IC_{50} value of Erlotinib and Osimertinib in the parental, erlotinib resistant (ER) and Osimertinib resistant (OR) NSCLC cells with their EGFR mutation status. IC_{50} values are calculated using MTT cell survival assays and statistical analysis was performed using 2 tailed t-test with $p<0.05$ as a significance value.

TABLE S2. DEMOGRAPHICS FOR NSCLC PATIENTS WHOSE LUNG TUMOR TISSUES WERE USED FOR THE STUDY OF FTO EXPRESSION

Tumor Stage	Sex		Race			Age			Smoking Status			
	M	F	White	Black	Unknown	40-59	60-79	80+	Current	Former	Never	Unknown
Early	29	35	61	2	1	16	42	6	14	33	17	0
Late	24	27	44	2	5	11	36	4	11	33	5	2

Table S2. Demographics for the 64 early-stage and 51 late-stage NSCLC patients from whom NSCLC Tumor Tissues were used for the study of FTO expression. The demographic table shows the sex, race, age, smoking status and tumor subtypes of the patients chosen in the study. They were mostly Caucasian and African Americans and were above 40 years of age. The de-identified tumor sections used for this study have been taken from lung cancer patients prior to any treatment.
